# Evaluation of Seven Essential Oils as Seed Treatments against Seedborne Fungal Pathogens of *Cucurbita maxima*

**DOI:** 10.3390/molecules26082354

**Published:** 2021-04-18

**Authors:** Marwa Moumni, Mohamed Bechir Allagui, Kaies Mezrioui, Hajer Ben Amara, Gianfranco Romanazzi

**Affiliations:** 1 Department of Agricultural, Food and Environmental Sciences, Marche Polytechnic University, 60131 Ancona, Italy; m.moumni@staff.univpm.it (M.M.); kaismezrioui@yahoo.fr (K.M.); 2 Laboratory of Plant Protection, National Institute for Agronomic Research of Tunisia, University of Carthage, 2080 Ariana, Tunisia; allagui.bechir@gmail.com (M.B.A.); mzbenamara@gmail.com (H.B.A.)

**Keywords:** essential oils, *Cymbopogon citratus*, seedborne pathogens, squash, *Stagonosporopsis cucurbitacearum*

## Abstract

Essential oils are gaining interest as environmentally friendly alternatives to synthetic fungicides for management of seedborne pathogens. Here, seven essential oils were initially tested in vivo for disinfection of squash seeds (*Cucurbita maxima*) naturally contaminated by *Stagonosporopsis cucurbitacearum*, *Alternaria alternata*, *Fusarium fujikuro*, *Fusarium solani*, *Paramyrothecium roridum*, *Albifimbria verrucaria*, *Curvularia spicifera*, and *Rhizopus stolonifer*. The seeds were treated with essential oils from *Cymbopogon citratus*, *Lavandula dentata*, *Lavandula hybrida*, *Melaleuca alternifolia*, *Laurus nobilis*, and *Origanum majorana* (#1 and #2). Incidence of *S. cucurbitacearum* was reduced, representing a range between 67.0% in *L. nobilis* to 84.4% in *O. majorana* #2. Treatments at 0.5 mg/mL essential oils did not affect seed germination, although radicles were shorter than controls, except with *C. citratus* and *O*. *majorana* #1 essential oils. Four days after seeding, seedling emergence was 20%, 30%, and 10% for control seeds and seeds treated with *C. citratus* essential oil (0.5 mg/mL) and fungicides (25 g/L difenoconazole plus 25 g/L fludioxonil). *S*. *cucurbitacearum* incidence was reduced by ~40% for plantlets from seeds treated with *C. citratus* essential oil. These data show the effectiveness of this essential oil to control the transmission of *S. cucurbitacearum* from seeds to plantlets, and thus define their potential use for seed decontamination in integrated pest management and organic agriculture.

## 1. Introduction

Disease management is one of the biggest challenges for quantity and quality of crop production. As pathogen infections can be difficult to control, early treatments with antimicrobial compounds are often used to provide disease prevention. *Cucurbita* spp. can be affected by many fungal diseases, such as gummy stem blight (caused by *Stagonosporopsis cucurbitacearum*), *Fusarium* fruit rot (caused by *Fusarium solani* f. sp. *cucurbitae*), *Alternaria* leaf spot (caused by *Alternaria alternata*, *Alternaria cucumerina*) [1,2,3,4], and bacterial spot of pumpkin (caused by *Xanthomonas cucurbitae*) [5], along with viral diseases, including squash mosaic virus [6]. All of these pathogens that are responsible for the main diseases on cucurbits can be carried on seeds. This association between seeds and pathogens is an important means for the pathogens to spread on a large scale and a way to guarantee their survival in nature [7,8,9]. Seedborne pathogens can limit the production of many crops and can result in severe economic losses to growers [10,11,12,13].

Almost 90% of the world’s food crops are grown from seed [14], and so sowing healthy seeds is essential to improve crop yields and increase food production [15]. With the need to guarantee high-quality seeds, seed treatments with antimicrobials represent a crucial and important step for reduction of seed infections [16]. Integrated pest management strategies are designed to provide environmentally sound and economically feasible alternatives for seedborne disease management [17]. These strategies are needed to minimize the inoculum of potential pathogens on seeds, and they draw on management components that are currently available to farmers, or can be made available in the near future [18].

Several studies have reported in vitro activities of essential oils and plant extracts against fungal and bacterial plant pathogens [19,20,21,22]. However, few have focused on in vivo activities of essential oils against seedborne fungi [23,24]. However, with many pathogens managed using fungicides, some of them have developed resistance to various fungicides that were previously very effective [25,26,27,28,29]. *Stagonosporopsis* spp. are devastating fungal pathogens of cucurbits and can lead to severe yield losses. These pathogens are major seedborne fungi of pumpkin and the dominant fungal cause of pumpkin seedling gummy stem blight [11,30]. No commercial cultivars with resistance to gummy stem blight are available, and so this disease has been frequently managed by fungicides application. As a result, several fungicides have lost their effectiveness against this disease following the development of resistance by *S. cucurbitacearum* [28,31]. Therefore, the development of alternative strategies to the use of synthetic fungicides for seed treatments is essential for both integrated pest management and organic agriculture. 

Among the alternatives to synthetic fungicides, essential oils have gained interest for such plant disease management [32]. Indeed, lemongrass essential oil (*Cymbopogon citratus*) was shown to provide 100% inhibition of mycelial growth of *S. cucurbitacearum* [22,33]. Moreover, Dalcin et al. [34] reported that treatment with a low concentration of lemongrass essential oil (<0.3%) reduced the severity of *S. cucurbitacearum* infections in melon plants. 

The aims of the present study were to investigate the effectiveness of seed treatments with seven essential oils against the major seedborne fungi of squash. Furthermore, we investigated whether the treatment of seeds with *C. citratus* essential oil can control the transmission of *S. cucurbitacearum* from seeds to plantlets.

## 2. Results

### 2.1. Efficacy of Essential Oils in the Control of Seed Infections

**Figure 1:**
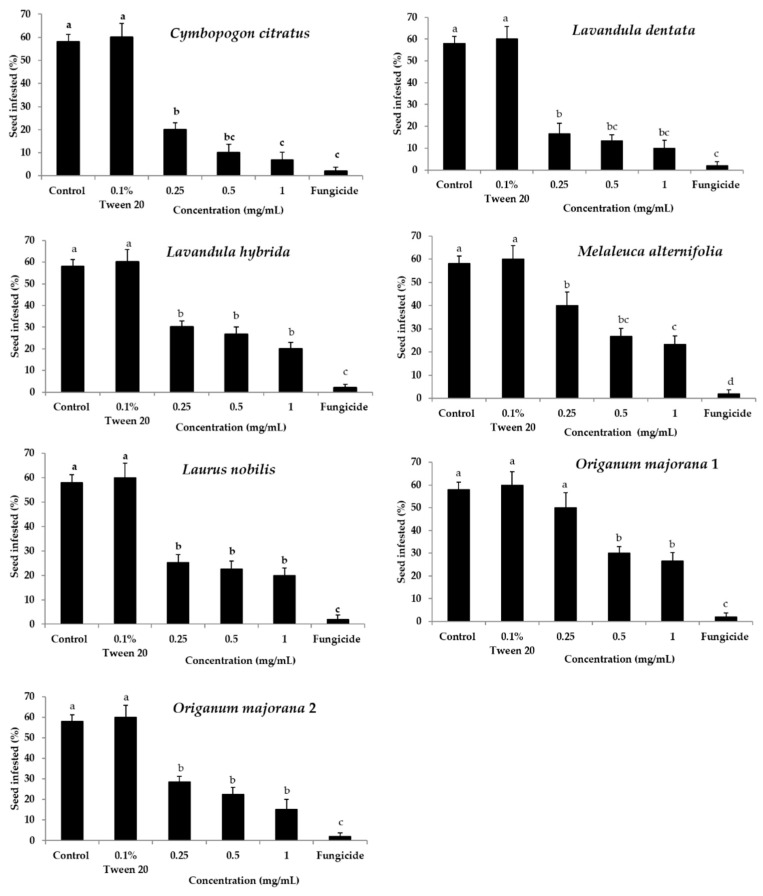
Incidence of infected seeds after treatments with sterile distilled water (negative control), 0.1% Tween 20, three concentrations of the seven essential oils (as indicated), and the fungicides combination (25 g/L difenoconazole plus 25 g/L fludioxonil; positive control), assayed using the blotter method. Data are means ± standard error (*n* = 10; 10 seeds/treatment/concentration). Means with different letters are significantly different (*p* ≤ 0.05; Fisher’s LSD tests).

Seven essential oils were used for seed treatments, initially at the three concentrations of 0.25, 0.5, and 1 mg/mL essential oils, using the standard blotter assay. The results show that these essential oils significantly reduced the incidence of infected seeds relative to the negative controls (Figure 1). The *Cymbopogon citratus* and *Lavandula dentata* essential oils at 0.5 mg/mL and 1 mg/mL were highly effective against incidence of infected seeds (ranged from 77.0–88.5%), compared to the similar levels achieved by the fungicide treatment (96.5%). Moreover, the incidence of infected seeds was significantly reduced by treatments at 1 mg/mL with *Lavandula hybrida*, *Melaleuca alternifolia*, *Laurus nobilis*, and the two *Origanum majorana* (#1 and #2) essential oils, by 66.5%, 59.8%, 65.5%, 54.1%, and 73.5%, respectively. Furthermore, the effects on the incidence of seed infections of these seven essential oils at 0.5 mg/mL and 1 mg/mL were not significantly different across the essential oils (*p* > 0.05).

### 2.2. Effects of 0.5 mg/mL Essential Oils on Seed Germination, Radicle Length, and Individual Fungal Seed Infections

The effects of 0.5 mg/mL essential oils were then studied in terms of seed germination and radicle length, and for the seed infections by the individual fungi in the blotter assays. 

From the initial data in Figure 1 and considering the rates of the individual fungal infections of the seeds from the blotter assays in Table 1, it can be seen that the treatments with the essential oils did protect against the potential for seedborne infections compared with the controls. As shown in Table 1, in the control, seeds the pathogen *S. cucurbitacearum* was frequently revealed to be carried by the seeds in the blotter assay (Table 1, 16.1%); this incidence rate was significantly reduced for this fungus for all of the seed treatments with the essential oils (overall mean incidence, 4.4%; overall inhibition, ~73%). In addition, the most effective of the essential oils were *O. majorana #2* and *M. alternifolia*, which significantly reduced seed infections by *S. cucurbitacearum* by 84.5% and 75.8%, respectively. These effects were also not significantly different compared to the seed treatment with the fungicides (98.8%). For the seeds that carried *A. alternata*, there were significant reductions for the seed treatments by the seven essential oils that ranged from 71.0% (*O. majorana #1*) to 89.3% (*L. dentata* and *L. nobilis*), again with no significant differences between the seeds treated with the essential oils and the fungicides. *Fusarium fujikuroi* was the most frequent fungus on the control seeds (incidence 22.6%) and considerably significantly reduced by the essential oils, in particular with the *C. citratus* and *L. dentata* essential oil treatments, by 76.6% and 89.4%, respectively, with these treatments also showing no significant differences compared to the seeds treated with the fungicides. All of the essential oils were also significantly effective against *F. solani* (except *L. hybrida*, at a non-significant 34.8% inhibition), with reductions of seed infections from 89.1–100%. The incidence of *Paramyrothecium roridum* was also significantly reduced for all of the seeds treated with the essential oils. Again, these effects were not significantly different between the essential oils, where the most effective inhibition appeared to be with *M. alternifolia* (87.9%), *L. dentata* (82.8%), and *O. majorana #1* (82.8%), with this fungus completely removed by the fungicides. The incidence of *Albifimbria verrucaria* on the seeds was significantly reduced by the *C. citratus*, *L. hybrida*, *L. nobilis*, and *O. majorana #2* essential oils, by 84.9%, 93.9%, 69.7%, and 69.7%, respectively. Instead, the inhibition by the remaining essential oils did not reach significance over the control (*L. dentata*, *M. alternifolia*, and *O. majorana #1*, all at 48.5%). For the seeds infected by *Curvularia spicifera*, there were reductions across these seven essential oils from 63.6% (*O. majorana #1*) to 100% (*L. dentata*), with relatively low, but not significantly different, inhibition seen for the fungicides (69.7%). *Rhizopus stolonifer* was significantly reduced by similar amounts in all of the seeds treated with the essential oils. The most effective here appeared to be *L. dentata* (77.4%) and *C. citratus* (74.4%), and the least effective were the two *O. majorana* essential oils (*#1* and *#2*: 50.6%, 49.4%, respectively); the fungicides showed 93.9% inhibition here.

The effects of the treatments with the seven essential oils at 0.5 mg/mL on seed germination and radicle length are also shown in Table 1. No significant differences in germination rates were seen between the control seeds and the seeds treated with the different essential oils. On the other hand, the germination of the seeds treated with the fungicide combination (25 g/L difenoconazole and 25 g/L fludioxonil) was significantly reduced, by 11.5%, compared to the control. Moreover, for the radicle length, these data showed that the *C. citratus* and *O. majorana #1* essential oils did not have any significant effects compared with the control. However, the radicle lengths of the seeds treated with the *L. dentata*, *L. hybrida*, *M. alternifolia*, *L. nobilis*, and *O. majorana #2* essential oils were significantly decreased by 28.2%, 23.4%, 34.5%, 25.8%, and 27.8%, respectively. On the contrary, compared to the control, the radicle length of the seeds treated with the fungicides was significantly increased, by 24.4%.

### 2.3. Effects of Cymbopogon citratus Essential Oil on Plantlets

**Figure 2:**
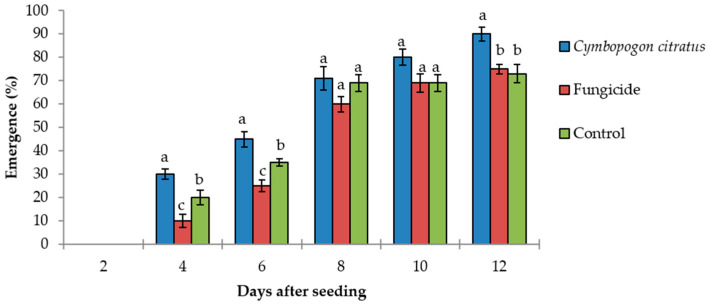
The effects of 0.5 mg/mL *Cymbopogon citratus* essential oil and fungicides (25 g/L difenoconazole plus 25 g/L fludioxonil) on seedling emergence. Control, seeds immersed in 0.1% Tween 20. Data are means ± standard error (*n* = 5; 10 seeds/treatment). Means with different letters are significantly different (*p* ≤ 0.05; Fisher’s LSD tests).

Seed treatments with 0.5 mg/mL *C. citratus* essential oil significantly increased the seedling emergence (*p* < 0.002) in comparison to the control and the fungicides treatments. Four days after sowing, the seeds treated with the *C. citratus* essential oil showed 30% emergence, while the control treatment (0.1% Tween 20) and the fungicides treatment showed 20% and 10% emergence, respectively. In particular, these data demonstrated that the seeds treated with the fungicides took longer to emerge (Figure 2).

**Figure 3:**
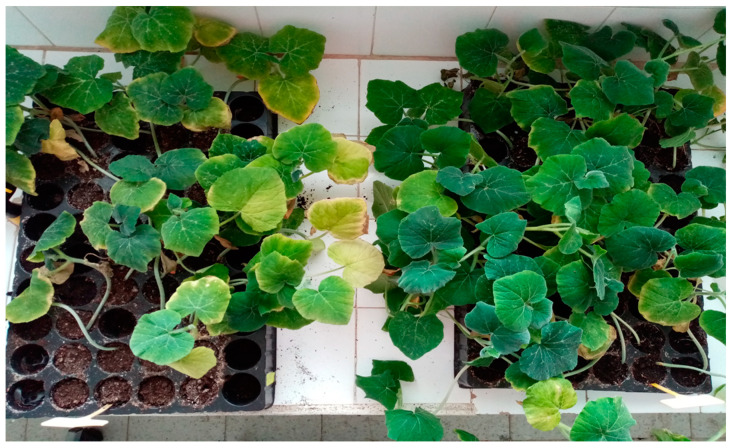
Gummy stem blight symptoms on squash plantlets. Control (**left**) and seeds treated with 0.5 mg/mL *Cymbopogon citratus* essential oil (**right**) 30 days from seeding, with plants kept at room temperature (22 ± 2 °C).

Forty days after seeding, compared to the control, these seed treatments with the *C. citratus* essential oil and the fungicides were effective for significant improvements in the plantlet lengths (23.3 cm vs. 30.5 cm, 30.2 cm, respectively; Table 2). These seeds treated with the *C. citratus* essential oil also resulted in significant reduction of *S. cucurbitacearum* on the plantlets (39.9%; *p* < 0.001) compared to the control (Table 2; Figure 3). In this assessment, the infections of the plantlet leaves, stems, and roots with *S. cucurbitacearum* were not significantly different between the *C. citratus* essential oil and fungicide treatments, which both showed significant improvements over the control. 

## 3. Discussion

Seeds are critical for the production of viable crops. The use of pathogen-free seeds is generally recommended as the primary management strategy for the generation of healthy plant populations and for good harvest yields [13,18,35]. Integrated pest management strategies can provide more environmentally sound and economically feasible alternatives for the management of seedborne diseases. These strategies are needed to minimize the potential inoculum of pathogens being carried on seeds, by drawing on management components that are currently available to farmers, or can be made available in the near future [18]. However, there remains a lack of information about the effectiveness of seed treatments with essential oils against fungal diseases of *C. maxima*.

The present study was performed in a controlled environment where seven essential oils were shown to be effective against the main seedborne fungi when applied to squash seeds that were naturally infected. The seed treatments with the *M. alternifolia* and *O. majorana #2* essential oils showed excellent activities against the *S. cucurbitacearum* and *F. solani* pathogens, with *F. solani* completely inhibited. These high antifungal activities of these two essential oils in particular appear to be the result of the high content of the monoterpene alcohol terpinen-4-ol, which in previous studies has shown good antifungal activity against *Fusarium* spp. [36,37]. 

Antifungal properties of essential oils towards *Fusarium* spp. have been reported in a number of previous studies, for different laboratory media and plant materials [38,39,40]. Perczak et al. [41] reported that essential oils have great potential for inhibition of growth of *Fusarium* fungi on maize seeds. An *O. majorana* essential oil was shown to have antifungal activities against four seedborne fungi in rice: *Fusarium verticilliodies*, *Fusarium graminearum*, *Bipolaris oryzae*, and *Curvularia lunata* [42]. Van der Wolf et al. [43] showed that thyme, oregano, cinnamon, and clove essential oils can reduce fungi on cabbage seeds, using the blotter method. Riccioni et al. [24] reported the efficacy of a tea tree essential oil to reduce Ascochyta blight in pea seeds. Their seed treatments with *L. nobilis* and *L. dentata* essential oils showed significant reductions of *A. alternata*. These two essential oils were characterized by high eucalyptol as their main volatile component. Xu et al. [44] showed excellent antifungal activity of a *L. nobilis* essential oil when tested against *A. alternata*. In addition, Xu et al. [45] demonstrated that 0.5 mg/mL of their *L. nobilis* essential oil protected cherry tomatoes from infection with *A. alternata*. 

The *L. hybrida* essential oil used in the present study was characterized previously to have relatively high content of linalool, followed by linalyl acetate and camphor [22]. Here, the *L. hybrida* essential oil showed moderate activity against seed infections. There were significant reductions in the *A. verrucaria* and *A. alternata* seed infections, although no significant reduction for *F. solani* compared to control. Daferera et al. [46] reported that the main compounds in their *L. hybrida* essential oil were linalyl acetate and linalool, and this essential oil showed less inhibitory activity against *Botrytis cinerea* and *F. solani* compared to oregano and thyme essential oils. The main compounds of a *Salvia sclarea* essential oil were also reported as linalyl acetate and linalool, and this essential oil showed good inhibitory activity against *A. alternata* [47]. Indeed, Kishore et al. [48] showed that linalool had the lowest inhibitory activity against *A. alternata*, *C. lunata, Fusarium moniliforme*, *Fusarium pallidoroseum*, and *Fusarium udum*. The same essential oil of *C. citratus* was shown to have the greatest inhibition of the growth of *S. cucurbitacearum* and *A. alternata* [22]. This activity has been confirmed in vivo in the present study, with reduction of the incidence of infection by the seedborne fungi for the plantlets following treatment of the seeds with the *C. citratus* essential oil. Chen et al. [49], demonstrated the effectiveness of a *Cymbopogon nardus* (citronella) essential oil on *A. alternata* in in vitro and in vivo assays. Eke et al. [50] reported that a *C. citratus* essential oil protected common bean plantlets from infection by *F. solani*, both in the laboratory and a greenhouse. 

A normal germination rate of >85% was seen here for all of the treated seeds, except with the fungicides. This confirms that these seven essential oils at concentration of 0.5 mg/mL are not phytotoxic to squash seed germination. Orzali et al. [51] showed also that an *Origanum vulgare* essential oil did not affect germination rates of tomato seeds. However, the concentration of essential oil and the immersion time of the seeds in the treatments are very important factors for phytotoxicity, as they can affect seed vitality [52,53]. In addition, here the *C. citratus* essential oil showed no significant inhibition of germination while it significantly inhibited the seed contamination, and unlike other essential oils here, *C. citratus* did not show significant shortening of the radicle length compared to the control following seed germination. For these reasons, this essential oil was selected in the final stages of this study, to be tested on the emergence and growth of the squash plantlets, and the disease incidence of *S. cucurbitacearum* for these plantlets. 

Here, the seed treatment with 0.5 mg/mL *C. citratus* essential oil significantly increased the plantlet emergence, reduced the incidence of seedborne *S. cucurbitacearum* for the various parts of the plantlets, without any significant differences compared to the fungicides. Naveenkumar et al. [54] reported that for *Oryza sativa* (rice) seedlings, a *C. citratus* essential oil increased the seed germination, shoot length, root length, and vigor. Also, a *C. citratus* essential oil was shown to reduce the severity of *S. cucurbitacearum* infections in melon plants [34]. 

Therefore, the use of these seven essential oils might serve as alternatives to the use of synthetic fungicides for prevention and control of seedborne pathogens in commercially produced seeds. The results of this study on the seedborne fungi of squash are thus useful to go forward to set up strategies for an integrated pest management program. 

## 4. Materials and Methods

### 4.1. Collection of Squash Seed Samples

Twenty-nine seed samples were collected from three regions in Tunisia (Siliana: 35°57′28″ N, 9°32′57″ E; Bizerte: 37°03′25″ N, 10°03′43″ E; and Kasserine: 35°14′00″ N, 9°08′00″ E). These seed samples were extracted from asymptomatic and symptomatic squash fruit (*Cucurbita maxima*, local variety cv. Bjaoui). These samples were evaluated for incidence of seedborne fungal pathogens, especially the main fungi: *S*. *cucurbitacearum*, *A*. *alternata*, and *F*. *solani* [4]. All of these seed samples were mixed and stored in paper bags at 4 °C until use in the experiments. 

### 4.2. Essential Oils

The seven essential oils used in this study were provided by different laboratories (Table 3) and were initially evaluated under in vitro conditions against the main seedborne fungi of cucurbits (*S*. *cucurbitacearum*, and *A*. *alternata*) [22]. The main volatile compounds present in these essential oils were previously analyzed by gas chromatography–mass spectrometry [22] (Table 3).

### 4.3. Seed Treatments with the Essential Oils

Before the treatments, naturally contaminated seeds were surface sterilized in 1% sodium hypochlorite solution for 5 min, rinsed three times with sterilized distilled water, and air dried for 2 h on sterile paper towel in a laminar flow hood. For the seed treatments, the seven essential oils were dissolved in sterilized distilled water with 0.1% (*v*/*v*) Tween 20 (Sigma Aldrich, Steinheim, Germany), to obtain homogeneous emulsions. Three final concentrations for each essential oil were initially used to determine the most effective concentration: 0.25 mg/mL, 0.5 mg/mL, and 1 mg/mL essential oils. The naturally infected seeds were immersed in 40 mL of each concentration of the essential oils, and in the fungicides (Celest Extra 17%: 25 g/L difenoconazole + 25 g/L fludioxonil). In parallel, two negative controls were prepared of the seeds in sterile distilled water and in 0.1% Tween 20. These seed treatments were carried out for 6 h, with mixing every 30 min. Then, the seeds were dried on sterile blotter sheets overnight (12 h) at room temperature (25–28 °C). The treated seeds were placed in glass Petri dishes that each contained eight pieces of sterile blotter paper (diameter, 110 mm; Whatman no. 4 filter papers) that were moistened with 5 mL sterile distilled water. These were incubated for 14 days at 22 ± 2 °C with a 12/12 h dark/ultraviolet light photoperiod (TL-D 36W BLB 1SL, Philips, Dublin, Ireland). To determine number of infected seeds, all Petri dishes were examined under a stereomicroscope (M125; Leica Microsystems CMS, Wetzlar, Germany). For each concentration of the different essential oils, 100 seeds were treated and the experimental trial was repeated twice. 

To compare the efficacies of the different essential oils on the fungi of these naturally infecting seeds, the intermediary 0.5 mg/mL concentration was chosen. Here, 200 seeds were treated separately with each of the seven essential oils at 0.5 mg/mL, as described above. Seeds immersed in 0.1% Tween 20 were used as the control. After 14 days of incubation, fungi identification was carried out first by examination of the fungal fruiting bodies and the congregated mycelia and spores on the seeds under a stereomicroscope (M125; Leica Microsystems CMS, Wetzlar, Germany). Then, the individual spores, conidiophores, and pycnidia were examined under a microscope (DM 2500; Leica). The fungal species identification was based on the keys of Mathur and Kongsdal [15] and Moumni et al. [4]. Assessments were based on relative frequencies of seedborne fungi, the germination rates of the seeds and the length of the growing radicle.

### 4.4. Effects of Cymbopogon citratus Essential Oil on Emergence of Seedlings and Disease Incidence of S. cucurbitacearum of Squash Plantlets

The naturally contaminated seeds were treated separately with the *C. citratus* essential oil at 0.5 mg/mL and with the fungicides (25 g/L difenoconazole + 25 g/L fludioxonil). Tween 20 (0.1%) was used as the control. For each treatment, 50 seeds were used, according to the method described above, and the experimental trials were repeated twice. The treated seeds were sown on pasteurized soil in disinfected seedling trays. The seeds in the trays were incubated at room temperature (22 ± 2 °C). Seedling emergence was recorded based on the number of emerged seedlings every 2 days, up to 14 days after sowing. At 14 days after sowing, the dead and germinated seedlings were recorded. Then 40 days after sowing, the lengths of the plantlets were measured and recorded. At the same time, 30 symptomatic and asymptomatic plantlets from each treatment were collected randomly and sliced into their three major parts—the leaves, stems, and roots—using a flame-sterilized scalpel, to determine the incidence of *S. cucurbitacearum* infection for each part. Each vegetative section was surface-disinfected in 1% sodium hypochlorite for 2 min, rinsed three times in sterile distilled water, and dried on sterile blotter paper. Each part was cut into pieces of about 2 mm, plated on potto dextrose agar, and assessed for fungal colony development after 7 days of incubation at 22 ± 2 °C. Morphological identification of the colonies was carried out to determine *S. cucurbitacearum* growth. The proportions of *S. cucurbitacearum* transmission (*Sc t*) we calculated based on Equation (1):

*Sc t* (%) = [Number of infected sections of plantlet/Total section of plantlet] × 100 (1)

### 4.5. Statistical Analysis

Analysis of variance was calculated using SPSS (version 20; IBM, Armonk, NY, USA). The data for incidence of infected seeds, germination rates, radicle lengths, plantlet infection, and plantlet lengths underwent analysis of variance (ANOVA). Means were compared using Fisher’s tests for protected least significant difference (LSD) at *p* ≤ 0.05. All of the trials were repeated at least twice, and the data are given as means ±standard error (SE). The following sample sets were used: 10 replicates of 10 seeds for the effects of the essential oils on disease incidence at three concentrations; 20 replicates of 10 seeds from each treatment to determine the effect of treatments on infected seeds at 0.5 mg/mL; and 5 replicates of 10 seeds from each treatment to determine the effect of *C. citratus* on emergence of seedling and disease incidence on the plantlets.

## 5. Conclusions

This study investigated the in vivo antifungal activities of seven essential oils towards seedborne pathogens of cucurbits. The results show that the essential oils are effective to reduce multiple pathogens on squash seeds. The *C*. *citratus* essential oil increased seedling emergence and reduced the incidence of *S. cucurbitacearum* in plantlets. 

Seed treatments with essential oils can contribute to effective management of seedborne fungi on cucurbits under integrated and organic agriculture. These strategies are needed to minimize the inoculum of potential pathogens on seeds, and can be available in the future. The success of the application of such nonchemical alternatives requires an integrated approach that involves the combination of multiple control strategies according to the localization of the pathogen on the seeds. These methods of alternative strategies now need to be further developed as relevant pest management tools for sustainable agricultural production.

## Figures and Tables

**Table 1:** Effects of the seven essential oils at 0.5 mg/mL and the fungicides on seed germination, radicle length, and incidence of seed infections by the individual fungi.

Treatment/ Essential Oil	Germination (%)	Radicle Length (cm)	Incidence of Seed Infection (%) ^c^							
			*S.c*.	*A.a*.	*F.f*.	*F.s*.	*P.r*.	*A.v*.	*C.s*.	*R.s*.
Control ^a^	85 ± 1.4 ^a^	20.9 ± 0.7 ^b^	16.1 ± 2.3 ^a^	9.3 ± 1.8 ^a^	22.6 ± 2.4 ^a^	4.6 ± 1.0 ^a^	5.8 ± 1.6 ^a^	3.3 ± 1.6 ^a^	3.3 ± 1.7 ^a^	16.4 ± 3.9 ^a^
*C. citratus*	86 ± 1.9 ^a^	21.0 ± 0.9 ^b^	4.5 ± 0.9 ^b^	1.1 ± 0.5 ^c^	5.3 ± 1.2 ^c,d^	0.5 ± 0.3 ^b^	1.7 ± 0.6 ^b^	0.5 ± 0.3 ^b^	0.9 ± 0.4 ^b^	4.2 ± 1.4 ^b,c^
*L. dentata*	86 ± 1.7 ^a^	15.0 ± 0.7 ^c^	5.2 ± 1.2 ^b^	1.0 ± 0.4 ^c^	2.4 ± 0.8 ^c,d^	0.5 ± 0.5 ^b^	1.0 ± 0.0 ^b^	1.7 ± 0.6 ^a,b^	0.0 ± 0.0 ^b^	3.7 ± 1.1 ^b,c^
*L. hybrida*	85 ± 2.2 ^a^	16.0 ± 0.6 ^c^	4.3 ± 0.9 ^b^	2.3 ± 0.8 ^c^	8.0 ± 1.4 ^c^	3.0 ± 1.6 ^a^	2.3 ± 0.8 ^b^	0.2 ± 0.2 ^b^	1.0 ± 0.6 ^b^	6.1 ± 1.9 ^b,c^
*M. alternifolia*	85 ± 2.4 ^a^	13.7 ± 0.7 ^c^	3.9 ± 1.2 ^b,c^	1.7 ± 0.6 ^c^	7.9 ± 1.3 ^c^	0.0 ± 0.0 ^b^	0.7 ± 0.4 ^b^	1.7 ± 0.8 ^a,b^	0.2 ± 0.2 ^b^	7.6 ± 2.0 ^b,c^
*L. nobilis*	86 ±1.9 ^a^	15.5 ± 0.6 ^c^	5.3 ± 1.1 ^b^	1.0 ± 0.4 ^c^	9.3 ± 1.4 ^b,c^	0.5 ± 0.3 ^b^	1.2 ± 0.5 ^b^	1.0 ±0.4 ^b^	0.7 ± 0.4 ^b^	6.3 ± 1.8 ^b,c^
*O. majorana*	87 ± 1.5 ^a^	20.5 ± 0.9 ^b^	5.0 ± 1.3 ^b^	2.7 ± 0.7 ^c^	8.7 ± 1.6 ^b,c^	0.5 ± 0.3 ^b^	1.0 ± 0.0 ^b^	1.7 ± 0.6 ^a,b^	1.2 ± 0.5 ^b^	8.1 ± 1.9 ^b^
*O. majorana*	85 ± 1.4 ^a^	15.1 ± 0.7 ^c^	2.5 ± 0.7 ^b,c^	1.2 ± 0.5 ^c^	10.1 ± 1.6 ^b^	0.0 ± 0.0 ^b^	1.7 ± 0.6 ^b^	1.0 ± 0.4 ^b^	0.3 ± 0.3 ^b^	8.3 ± 2.4 ^b^
Fungicides ^b^	75 ± 2.2 ^b^	26.0 ± 1.1 ^a^	0.2 ± 0.2 ^c^	0.0 ± 0.0 ^c^	1.21 ± 0.5 ^d^	0.0 ± 0.0 ^b^	0.0 ± 0.0 ^b^	0.0 ± 0.0 ^b^	1.0 ± 0.0 ^b^	1.0 ± 0.0 ^c^
Significance (*p*)	0.001	<0.001	<0.001	<0.001	<0.001	<0.001	0.002	0.025	0.032	<0.001

^a^ Seeds immersed in 0.1% Tween 20. ^b^ 25 g/L difenoconazole + 25 g/L fludioxonil. ^c^ *S.c.*, *Stagonosporopsis cucurbitacearum*; *A.a.*, *Alternaria alternata*; *F.f.*, *Fusarium fujikuroi*; *F.s.*, *Fusarium solani*; *P.r.*, *Paramyrothecium roridum*; *A.v.*, *Albifimbria verrucaria*; *C.s.*, *Curvularia spicifera*; *R.s.*, *Rhizopus stolonifer*. Data are means ± SE (*n* = 20; 10 seeds/treatment). Means with different letters are significantly different between treatments (down columns) (*p* ≤ 0.05; Fisher’s LSD).

**Table 2:** Effects of 0.5 mg/mL *Cymbopogon citratus* essential oil and the fungicides (25 g/L difenoconazole, 25 g/L fludioxonil) on squash plantlets and their incidence of *Stagonosporopsis cucurbitacearum* after 40 days of seeding at room temperature (22 ± 2 °C).

Treatment	Plantlet		Disease Incidence of *Stagonosporopsis cucurbitacearum* on the Plantlets (%)		
	Length (cm)	Infection (%)	Leaves	Stems	Roots
Control ^a^	23.3 ± 1.2 ^a^	49.9 ± 2.9 ^c^	30.3 ± 5.2 ^b^	43.0 ± 4.4 ^b^	43.1 ± 3.2 ^b^
*C. citratus*	30.5 ± 1.1 ^b^	30.0 ± 4.4 ^b^	18.7 ± 3.3 ^a^	28.3 ± 4.5 ^a^	18.1 ± 4.3 ^a^
Fungicide ^b^	30.2 ± 1.2 ^b^	16.8 ± 1.9 ^a^	9.7 ± 1.6 ^a^	17.3 ± 1.9 ^a^	17.0 ± 2.1 ^a^

^a^ Seeds immersed in 0.1% Tween 20. ^b^ 25 g/L difenoconazole + 25 g/L fludioxonil. Data are means ± SE (*n* = 5; 10 seeds/treatment). Data with different letters are significantly different between treatments (down columns) (*p* ≤ 0.05; Fisher’s LSD).

**Table 3:** Details of the seven essential oils used in this study, including the two main volatile constituents defined through previous gas chromatography–mass spectrometry analysis [22].

Species	Common Name	Source	Two Main Components	
			Compound	(%)
*Cymbopogon citratus*	Lemongrass	CRRHAB	α-Citral	51.6
			𝛽-Citral	26.0
*Lavandula dentata*	Lavender	CRRHAB	Eucalyptol	63.5
			β-Selinene	4.1
*Lavandula hybrida*	Lavandin	FLORA s.r.l.	Linalool	33.7
		(Batch N° 161808)	Camphor	9.3
*Melaleuca alternifolia*	Tea tree	FLORA s.r.l.	Terpinen-4-ol	41.1
		(Batch N° 161960)	γ-Terpinene	16.0
*Laurus nobilis*	Bay laurel	INAT	Eucalyptol	47.9
			𝛼-Terpinyl acetate	10.2
*Origanum majorana*	Marjoram#1	INAT	Terpinen-4-ol	32.4
			γ-Terpinene	12.6
*Origanum majorana*	Marjoram#2	CRRHAB	Terpinen-4-ol	50.1
			p-Cymene	17.8

CRRHAB, Biopesticides Laboratory, Regional Centre for Research in Horticulture and Organic Agriculture, Sousse, Tunisia. FLORA s.r.l., Lorenzana, Pisa, Italy. INAT, Medicinal Plants Laboratory, National Institute of Agronomy of Tunisia, Tunisia.

## Data Availability

The data presented in this study are available on request from the corresponding author.

## Sample Availability

Samples of the compounds are available from the authors.
